# (*Z*)-3-(2-Methoxy­anilino)-1-phenyl­but-2-en-1-one

**DOI:** 10.1107/S1600536810004460

**Published:** 2010-02-10

**Authors:** Li-Ping Zhang, Li-Yun Qi, Yun Fang

**Affiliations:** a School of Chemical and Materials Engineering, Jiangnan University, 1800 Lihu Road, Wuxi 214122, Jiangsu, People’s Republic of China

## Abstract

In the title compound, C_17_H_17_NO_2_, the dihedral angle between the two benzene rings is 55.2 (2)°. The meth­oxy group is slightly twisted away from the aniline ring [dihedral angle = 10.3 (2)°]. An intra­molecular N—H⋯O inter­action is present. In the crystal, the mol­ecules are linked into a three-dimensional supra­molecular network through two sets of C—H⋯π inter­actions.

## Related literature

For the use of β-enamino ketones as inter­mediates for the synthesis of natural therapeutic and biologically active analogues, see:Azzaro *et al.* (1981 ▶); Dannhardt *et al.* (1998 ▶); Boger *et al.* (1989 ▶); Wang *et al.* (1982 ▶). For the synthesis of β-enamino ketones, see: Greenhill *et al.* (1977 ▶); Elassar & El-Khair (2003 ▶); Zhang *et al.* (2006 ▶).
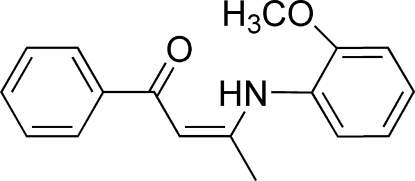


## Experimental

### 

#### Crystal data

C_17_H_17_NO_2_
*M* *_r_* = 267.32
Tetragonal, 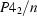

*a* = 19.125 (2) Å
*c* = 7.9993 (19) Å
*V* = 2925.9 (8) Å^3^
*Z* = 8
Mo *K*α radiation
μ = 0.08 mm^−1^
*T* = 294 K
0.20 × 0.12 × 0.10 mm

#### Data collection

Bruker SMART CCD area-detector diffractometer
Absorption correction: multi-scan (*SADABS*; Sheldrick, 1996 ▶) *T*_min_ = 0.984, *T*_max_ = 0.992
15499 measured reflections
3001 independent reflections
1457 reflections with *I* > 2σ(*I*)
*R*_int_ = 0.067

#### Refinement

*R*[*F* ^2^ > 2σ(*F* ^2^)] = 0.042
*wR*(*F* ^2^) = 0.115
*S* = 1.02
3001 reflections
184 parameters
H-atom parameters constrained
Δρ_max_ = 0.13 e Å^−3^
Δρ_min_ = −0.12 e Å^−3^

### 

Data collection: *SMART* (Bruker, 1998 ▶); cell refinement: *SAINT* (Bruker, 1999 ▶); data reduction: *SAINT*; program(s) used to solve structure: *SHELXS97* (Sheldrick, 2008 ▶); program(s) used to refine structure: *SHELXL97* (Sheldrick, 2008 ▶); molecular graphics: *SHELXTL* (Sheldrick, 2008 ▶); software used to prepare material for publication: *SHELXTL*.

## Supplementary Material

Crystal structure: contains datablocks global, I. DOI: 10.1107/S1600536810004460/bv2137sup1.cif

Structure factors: contains datablocks I. DOI: 10.1107/S1600536810004460/bv2137Isup2.hkl

Additional supplementary materials:  crystallographic information; 3D view; checkCIF report

## Figures and Tables

**Table 1:** Hydrogen-bond geometry (Å, °) *Cg*1 is the centroid of the benzene ring. C13 is the nearest aromatic atom to H16.

*D*—H⋯*A*	*D*—H	H⋯*A*	*D*⋯*A*	*D*—H⋯*A*
N1—H1⋯O2	0.86	1.91	2.639 (2)	139
C3—H3⋯*Cg*1^i^	0.93	2.79	3.725 (2)	153
C16—H16⋯π(C13)^ii^	0.93	2.78	3.688 (4)	167

Symmetry codes: (i) 
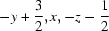
; (ii) 
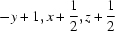
.

**Fig. 1.**
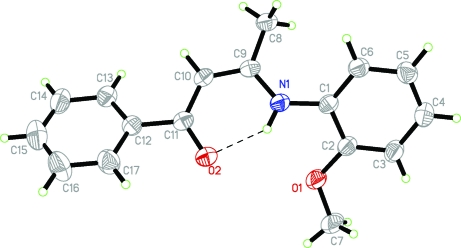
The molecular structure of the title compound, showing 30% probability displacement ellipsoids.

**Fig. 2.**
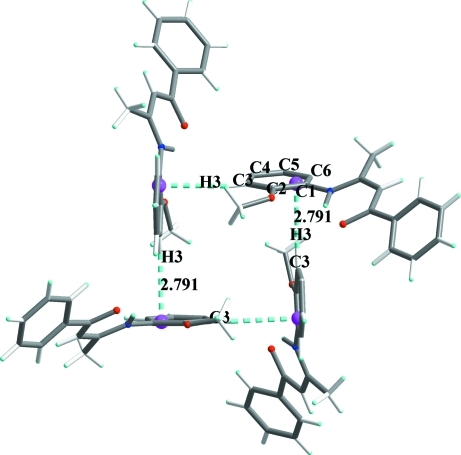
The intermolecular C—H···π interactions between aromatic rings of adjacent molecules.

**Fig. 3.**
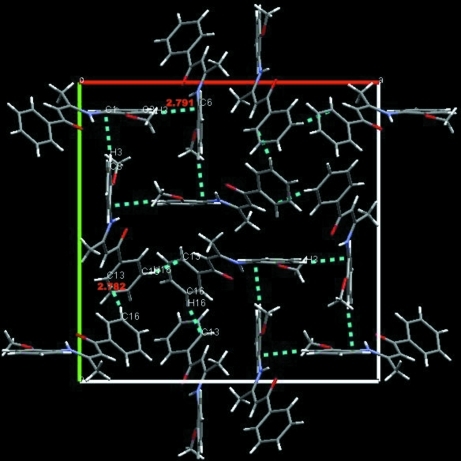
The molecular packing of the title compound, showing the C—H···π three-dimensional supramolecular network.

## supplementary crystallographic information

### Comment

β-Enamino ketones have attracted much interest because they are versatile intermediates for the synthesis of natural therapeutic and biologically active analogues including taxo anticonvulsivant (Azzaro *et al.*, 1981), anti-inflammatory (Dannhardt *et al.*, 1998) and antitumor agents (Boger *et al.*, 1989) as well as quinolone antibacterials (Wang *et al.*, 1982) It is therefore not surprising that many synthetic methods have been developed for the preparation of these compounds (Greenhill *et al.*,1977; Elassar *et al.*, 2003). In our developing new environmental friendly methodologies (Zhang *et al.*, 2006) for the preparation of β-enamino ketones, we synthesized the title compound (I) (Fig.1), the synthesis and crystal structure of which are reported here.

In the title compound, the ring C1—C6 forms dihedral angles of 10.3 (2)° and 124.8 (2)°, respectively, with the C7—O1—C2 methoxy group and the C12—C17. The bond lengths C10—C11 [1.417 (3) Å] and N1—C9 [1.349 (2) Å] are slightly shorter than corresponding C11—C12 [1.497 (3) Å], and N1—C1[1.404 (2) Å], indicating a weak electron delocalization.

In the crystal, each four centrosymmetry related molecules are linked by C3—H1—*Cg*1 interactions into a four-leaves windmill (Fig.2), which are further linked into a three-dimensional supramolecular network by C16—H16—*Cg*2 interactions(Fig.3). The C—H···π distance is 2.79Å for C3—H1—*Cg*1 (*Cg*1: C1/C2—C3), with an angle of 152.8 (2)° and 2.78%A for C16—H16—*Cg*13 with an angle of 166.5 (3)°.

### Experimental

A mixture of the 1-phenylbutane-1,3-dione (5 mmol), 2-methoxybenzenamine (5 mmol) and InBr_3_ (0.05 mmol) was stirred at room temperature for 1 h. After completion of the reaction, the reaction mixture was diluted with H_2_O (10 ml) and extracted with EtOAc (210 ml). The combined organic layers were dried, concentrated, purified by column chromatography on SiO_2_ with ethyl acetate-cyclohexane (2: 8). A pale yellow solid was obtained, with a yield of 83%. mp 92–93οC; IR (neat):ν 3006, 1608, 1477, 1461, 1373, 1284,1119,1024, 748 cm^-1^; ^1^H NMR(CDCl_3_, 300 MHz): δ 2.14(s, 3H), 3.90(s, 3H), 5.91(s, 1H), 6.92–6.97(d, 2H,Ar—H), 7.17–7.22(m, 2H,Ar—H), 7.41–7.45(m, 3H,Ph), 7.91–7.94 (m, 2H, Ph), 12.87 (br s, 1H, NH). ^13^C NMR(CDCl_3_, 75 MHz): δ 20.4, 55.8, 94.4,111.4, 120.4, 125.3, 126.6, 127.1,128.1, 130.7,140.2,153.0, 162.3, 188.5. ESI-MS: 268(*M*+1)^+^ Anal. Calcd for C_17_H_17_NO_2_: C,76.38; H,6.41;N,5.24. Found:*C*,76.56; H,6.28; N,5.35.

Single crystals suitable for X-ray diffraction study were obtained from ethyl acetate-cyclohexane by slow evaporation at room temperature.

### Refinement

H atoms were placed in geometrically idealized positions and constrained to ride on their parent atoms, with N—H = 0.86 Å, C—H = 0.93–0.97 Å, and *U*_iso_(H) = 1.5*U*_eq_(CH_3_)or 1.2*U*_eq_(C,*N*). Each methyl group was allowed to rotate freely about its C—C bond.

### Crystal data

**Table:**

C_17_H_17_NO_2_	*D*_x_ = 1.214 Mg m^−^^3^
*M**_r_* = 267.32	Mo *K*α radiation, λ = 0.71073 Å
Tetragonal, *P*4_2_/*n*	Cell parameters from 2401 reflections
Hall symbol: -P 4bc	θ = 2.8–21.2°
*a* = 19.125 (2) Å	µ = 0.08 mm^−^^1^
*c* = 7.9993 (19) Å	*T* = 294 K
*V* = 2925.9 (8) Å^3^	Block, colorless
*Z* = 8	0.20 × 0.12 × 0.10 mm
*F*(000) = 1136	

### Data collection

**Table:**

Bruker SMART CCD area-detector diffractometer	3001 independent reflections
Radiation source: fine-focus sealed tube	1457 reflections with *I* > 2σ(*I*)
graphite	*R*_int_ = 0.067
ω scans	θ_max_ = 26.4°, θ_min_ = 1.5°
Absorption correction: multi-scan (*SADABS*; Sheldrick, 1996)	*h* = −22→23
*T*_min_ = 0.984, *T*_max_ = 0.992	*k* = −23→12
15499 measured reflections	*l* = −10→9

### Refinement

**Table:**

Refinement on *F*^2^	Secondary atom site location: difference Fourier map
Least-squares matrix: full	Hydrogen site location: inferred from neighbouring sites
*R*[*F*^2^ > 2σ(*F*^2^)] = 0.042	H-atom parameters constrained
*wR*(*F*^2^) = 0.115	*w* = 1/[σ^2^(*F*_o_^2^) + (0.0385*P*)^2^ + 0.433*P*] where *P* = (*F*_o_^2^ + 2*F*_c_^2^)/3
*S* = 1.02	(Δ/σ)_max_ < 0.001
3001 reflections	Δρ_max_ = 0.13 e Å^−^^3^
184 parameters	Δρ_min_ = −0.12 e Å^−^^3^
0 restraints	Extinction correction: *SHELXL*, Fc^*^=kFc[1+0.001xFc^2^λ^3^/sin(2θ)]^-1/4^
Primary atom site location: structure-invariant direct methods	Extinction coefficient: 0.0090 (9)

### Special details

**Table:**

Geometry. All e.s.d.'s (except the e.s.d. in the dihedral angle between two l.s. planes) are estimated using the full covariance matrix. The cell e.s.d.'s are taken into account individually in the estimation of e.s.d.'s in distances, angles and torsion angles; correlations between e.s.d.'s in cell parameters are only used when they are defined by crystal symmetry. An approximate (isotropic) treatment of cell e.s.d.'s is used for estimating e.s.d.'s involving l.s. planes.
Refinement. Refinement of *F*^2^ against ALL reflections. The weighted *R*-factor *wR* and goodness of fit *S* are based on *F*^2^, conventional *R*-factors *R* are based on *F*, with *F* set to zero for negative *F*^2^. The threshold expression of *F*^2^ > σ(*F*^2^) is used only for calculating *R*-factors(gt) *etc*. and is not relevant to the choice of reflections for refinement. *R*-factors based on *F*^2^ are statistically about twice as large as those based on *F*, and *R*- factors based on ALL data will be even larger.

### Fractional atomic coordinates and isotropic or equivalent isotropic displacement parameters (Å^2^)

**Table:**

	*x*	*y*	*z*	*U*_iso_*/*U*_eq_	
O1	0.66062 (7)	0.61242 (9)	0.03020 (19)	0.0750 (5)	
O2	0.50749 (8)	0.65620 (8)	0.22101 (19)	0.0700 (5)	
N1	0.53344 (8)	0.60548 (8)	−0.0795 (2)	0.0544 (5)	
H1	0.5444	0.6304	0.0061	0.065*	
C1	0.58654 (11)	0.59997 (10)	−0.1999 (3)	0.0518 (5)	
C2	0.65502 (11)	0.60408 (11)	−0.1391 (3)	0.0571 (6)	
C3	0.71061 (12)	0.60098 (13)	−0.2475 (3)	0.0709 (7)	
H3	0.7560	0.6032	−0.2064	0.085*	
C4	0.69923 (13)	0.59461 (13)	−0.4180 (3)	0.0743 (7)	
H4	0.7370	0.5917	−0.4908	0.089*	
C5	0.63235 (13)	0.59259 (12)	−0.4793 (3)	0.0676 (7)	
H5	0.6247	0.5889	−0.5938	0.081*	
C6	0.57633 (12)	0.59603 (11)	−0.3709 (3)	0.0595 (6)	
H6	0.5311	0.5957	−0.4133	0.071*	
C7	0.72721 (13)	0.62799 (16)	0.0979 (3)	0.0985 (9)	
H7A	0.7570	0.5878	0.0880	0.148*	
H7B	0.7223	0.6403	0.2137	0.148*	
H7C	0.7475	0.6664	0.0379	0.148*	
C8	0.44084 (11)	0.53573 (11)	−0.2157 (3)	0.0621 (6)	
H8A	0.4270	0.5660	−0.3056	0.093*	
H8B	0.4012	0.5092	−0.1786	0.093*	
H8C	0.4767	0.5044	−0.2539	0.093*	
C9	0.46824 (11)	0.57866 (10)	−0.0740 (2)	0.0493 (5)	
C10	0.42736 (10)	0.59017 (10)	0.0650 (3)	0.0532 (5)	
H10	0.3825	0.5715	0.0647	0.064*	
C11	0.44829 (11)	0.62827 (10)	0.2084 (3)	0.0535 (5)	
C12	0.39786 (11)	0.63783 (11)	0.3497 (3)	0.0548 (6)	
C13	0.34395 (11)	0.59189 (12)	0.3815 (3)	0.0654 (6)	
H13	0.3387	0.5524	0.3149	0.078*	
C14	0.29750 (13)	0.60378 (16)	0.5113 (3)	0.0844 (8)	
H14	0.2615	0.5722	0.5320	0.101*	
C15	0.30459 (18)	0.66161 (19)	0.6081 (4)	0.0987 (10)	
H15	0.2729	0.6702	0.6938	0.118*	
C16	0.35791 (18)	0.70697 (16)	0.5801 (4)	0.1039 (10)	
H16	0.3628	0.7462	0.6478	0.125*	
C17	0.40504 (14)	0.69538 (13)	0.4520 (3)	0.0807 (8)	
H17	0.4417	0.7265	0.4348	0.097*	

### Atomic displacement parameters (Å^2^)

**Table:**

	*U*^11^	*U*^22^	*U*^33^	*U*^12^	*U*^13^	*U*^23^
O1	0.0502 (10)	0.1141 (14)	0.0606 (10)	−0.0126 (9)	−0.0072 (8)	0.0100 (9)
O2	0.0586 (10)	0.0757 (11)	0.0757 (10)	−0.0146 (8)	0.0001 (8)	−0.0133 (9)
N1	0.0480 (11)	0.0562 (11)	0.0591 (11)	−0.0065 (9)	−0.0022 (9)	−0.0057 (9)
C1	0.0480 (13)	0.0461 (13)	0.0612 (15)	−0.0023 (10)	−0.0015 (12)	0.0043 (11)
C2	0.0490 (14)	0.0637 (15)	0.0586 (14)	−0.0054 (11)	−0.0027 (11)	0.0112 (12)
C3	0.0492 (14)	0.0939 (19)	0.0696 (17)	−0.0019 (12)	0.0022 (12)	0.0153 (14)
C4	0.0645 (17)	0.0842 (18)	0.0741 (18)	−0.0017 (13)	0.0158 (14)	0.0066 (14)
C5	0.0785 (18)	0.0670 (16)	0.0573 (14)	−0.0099 (13)	0.0032 (14)	0.0044 (12)
C6	0.0564 (14)	0.0610 (15)	0.0612 (15)	−0.0058 (11)	−0.0046 (12)	0.0036 (12)
C7	0.0568 (16)	0.162 (3)	0.0763 (18)	−0.0205 (17)	−0.0157 (14)	0.0046 (18)
C8	0.0584 (14)	0.0648 (14)	0.0633 (14)	−0.0089 (11)	−0.0098 (12)	0.0006 (12)
C9	0.0480 (12)	0.0414 (12)	0.0585 (13)	0.0022 (10)	−0.0109 (11)	0.0047 (10)
C10	0.0415 (12)	0.0545 (13)	0.0637 (14)	0.0003 (10)	−0.0038 (11)	0.0005 (12)
C11	0.0484 (13)	0.0459 (12)	0.0662 (14)	0.0020 (11)	−0.0060 (11)	0.0041 (11)
C12	0.0530 (14)	0.0537 (14)	0.0577 (13)	0.0089 (11)	−0.0037 (11)	0.0026 (11)
C13	0.0589 (15)	0.0682 (16)	0.0690 (15)	0.0030 (12)	0.0007 (13)	0.0041 (13)
C14	0.0638 (17)	0.104 (2)	0.0851 (18)	0.0042 (16)	0.0144 (15)	0.0109 (18)
C15	0.102 (2)	0.106 (3)	0.088 (2)	0.027 (2)	0.0247 (19)	−0.001 (2)
C16	0.132 (3)	0.086 (2)	0.094 (2)	0.007 (2)	0.028 (2)	−0.0225 (18)
C17	0.090 (2)	0.0689 (17)	0.0837 (18)	−0.0053 (14)	0.0130 (16)	−0.0124 (15)

### Geometric parameters (Å, °)

**Table:**

O1—C2	1.368 (2)	C8—C9	1.494 (3)
O1—C7	1.415 (2)	C8—H8A	0.9600
O2—C11	1.256 (2)	C8—H8B	0.9600
N1—C9	1.349 (2)	C8—H8C	0.9600
N1—C1	1.404 (2)	C9—C10	1.377 (3)
N1—H1	0.8600	C10—C11	1.417 (3)
C1—C6	1.384 (3)	C10—H10	0.9300
C1—C2	1.399 (3)	C11—C12	1.497 (3)
C2—C3	1.373 (3)	C12—C17	1.378 (3)
C3—C4	1.387 (3)	C12—C13	1.378 (3)
C3—H3	0.9300	C13—C14	1.385 (3)
C4—C5	1.370 (3)	C13—H13	0.9300
C4—H4	0.9300	C14—C15	1.357 (4)
C5—C6	1.380 (3)	C14—H14	0.9300
C5—H5	0.9300	C15—C16	1.357 (4)
C6—H6	0.9300	C15—H15	0.9300
C7—H7A	0.9600	C16—C17	1.383 (3)
C7—H7B	0.9600	C16—H16	0.9300
C7—H7C	0.9600	C17—H17	0.9300
			
C2—O1—C7	118.28 (17)	C9—C8—H8C	109.5
C9—N1—C1	131.48 (18)	H8A—C8—H8C	109.5
C9—N1—H1	114.3	H8B—C8—H8C	109.5
C1—N1—H1	114.3	N1—C9—C10	119.35 (19)
C6—C1—C2	118.6 (2)	N1—C9—C8	120.57 (19)
C6—C1—N1	125.47 (19)	C10—C9—C8	120.07 (18)
C2—C1—N1	115.77 (19)	C9—C10—C11	125.15 (19)
O1—C2—C3	124.7 (2)	C9—C10—H10	117.4
O1—C2—C1	115.07 (19)	C11—C10—H10	117.4
C3—C2—C1	120.2 (2)	O2—C11—C10	122.6 (2)
C2—C3—C4	120.2 (2)	O2—C11—C12	117.9 (2)
C2—C3—H3	119.9	C10—C11—C12	119.50 (19)
C4—C3—H3	119.9	C17—C12—C13	118.3 (2)
C5—C4—C3	120.1 (2)	C17—C12—C11	118.8 (2)
C5—C4—H4	120.0	C13—C12—C11	122.9 (2)
C3—C4—H4	120.0	C12—C13—C14	120.9 (2)
C4—C5—C6	119.9 (2)	C12—C13—H13	119.6
C4—C5—H5	120.1	C14—C13—H13	119.6
C6—C5—H5	120.1	C15—C14—C13	119.8 (3)
C5—C6—C1	121.0 (2)	C15—C14—H14	120.1
C5—C6—H6	119.5	C13—C14—H14	120.1
C1—C6—H6	119.5	C14—C15—C16	120.1 (3)
O1—C7—H7A	109.5	C14—C15—H15	119.9
O1—C7—H7B	109.5	C16—C15—H15	119.9
H7A—C7—H7B	109.5	C15—C16—C17	120.6 (3)
O1—C7—H7C	109.5	C15—C16—H16	119.7
H7A—C7—H7C	109.5	C17—C16—H16	119.7
H7B—C7—H7C	109.5	C12—C17—C16	120.2 (3)
C9—C8—H8A	109.5	C12—C17—H17	119.9
C9—C8—H8B	109.5	C16—C17—H17	119.9
H8A—C8—H8B	109.5		
			
C9—N1—C1—C6	34.4 (3)	N1—C9—C10—C11	−0.7 (3)
C9—N1—C1—C2	−150.6 (2)	C8—C9—C10—C11	178.21 (18)
C7—O1—C2—C3	8.6 (3)	C9—C10—C11—O2	0.7 (3)
C7—O1—C2—C1	−170.2 (2)	C9—C10—C11—C12	178.91 (18)
C6—C1—C2—O1	175.91 (19)	O2—C11—C12—C17	24.9 (3)
N1—C1—C2—O1	0.5 (3)	C10—C11—C12—C17	−153.5 (2)
C6—C1—C2—C3	−3.0 (3)	O2—C11—C12—C13	−155.7 (2)
N1—C1—C2—C3	−178.40 (19)	C10—C11—C12—C13	25.9 (3)
O1—C2—C3—C4	−178.0 (2)	C17—C12—C13—C14	1.0 (3)
C1—C2—C3—C4	0.8 (4)	C11—C12—C13—C14	−178.4 (2)
C2—C3—C4—C5	1.1 (4)	C12—C13—C14—C15	0.5 (4)
C3—C4—C5—C6	−0.8 (4)	C13—C14—C15—C16	−1.4 (4)
C4—C5—C6—C1	−1.4 (3)	C14—C15—C16—C17	0.8 (5)
C2—C1—C6—C5	3.3 (3)	C13—C12—C17—C16	−1.6 (4)
N1—C1—C6—C5	178.25 (19)	C11—C12—C17—C16	177.8 (2)
C1—N1—C9—C10	177.28 (19)	C15—C16—C17—C12	0.8 (4)
C1—N1—C9—C8	−1.6 (3)		

### Hydrogen-bond geometry (Å, °)

**Table:**

Cg1 is the centroid of the benzene ring. C13 is the nearest aromatic atom to H16.

**Table:**

*D*—H···*A*	*D*—H	H···*A*	*D*···*A*	*D*—H···*A*
N1—H1···O2	0.86	1.91	2.639 (2)	139
C3—H3···Cg1^i^	0.93	2.79	3.725 (2)	153
C16—H16···π(C13)^ii^	0.93	2.78	3.688 (4)	167

Symmetry codes: (i) −*y*+3/2, *x*, −*z*−1/2; (ii) −*y*+1, *x*+1/2, *z*+1/2.
